# Supplementary material for: Inverse stable isotope labeling (InverSIL) links predicted catecholate siderophore gene clusters to their products in diverse bacteria
Source: mBio. 2026 May 7;17(6):e03391-25. doi: 10.1128/mbio.03391-25 (PMC13251462; doi:10.1128/mbio.03391-25)
Supplement: Supplemental figures and tables — Figures S1-S20; Tables S1-S5. [file mbio.03391-25-s0001.pdf]

Supplemental Material for:

Inverse stable isotope labeling (InverSIL) links predicted catecholate siderophore gene clusters to their products in diverse bacteria

Jose Miguel D. Robes[1,2], Tashi C. E. Liebergesell[1,2], Victoria P. Medvedeva[1,2#], & Aaron W. Puri[1,2*]

[1]Department of Chemistry, University of Utah, Salt Lake City, Utah, USA
[2]Henry Eyring Center for Cell and Genome Science, University of Utah, Salt Lake City, Utah, USA

#Present address:
Victoria Medvedeva, Department of Chemistry, University of California, Irvine, Irvine, California, USA

*Corresponding author:
Aaron W. Puri
315 S 1400 E Rm 2020
Salt Lake City, UT 84112
USA
(801) 213-1408
a.puri@utah.edu

TABLE OF CONTENTS

SUPPLEMENTARY FIGURES 3
Figure S1 3
Figure S2. 4
Figure S3 5
Figure S4 7
Figure S5 8
Figure S6 9
Figure S7 10
Figure S8 11
Figure S9 12
Figure S10 14
Figure S11 15
Figure S12 17
Figure S13 18
Figure S14 19
Figure S15 20
Figure S16 21
Figure S17 22
Figure S18 23
Figure S19 24
Figure S20 25
SUPPLEMENTARY TABLES 26
Table S1........ 26
Table S2........ 27
Table S3........ 28
Table S4........ 29
Table S5........ 31
REFERENCES 32

SUPPLEMENTARY FIGURES

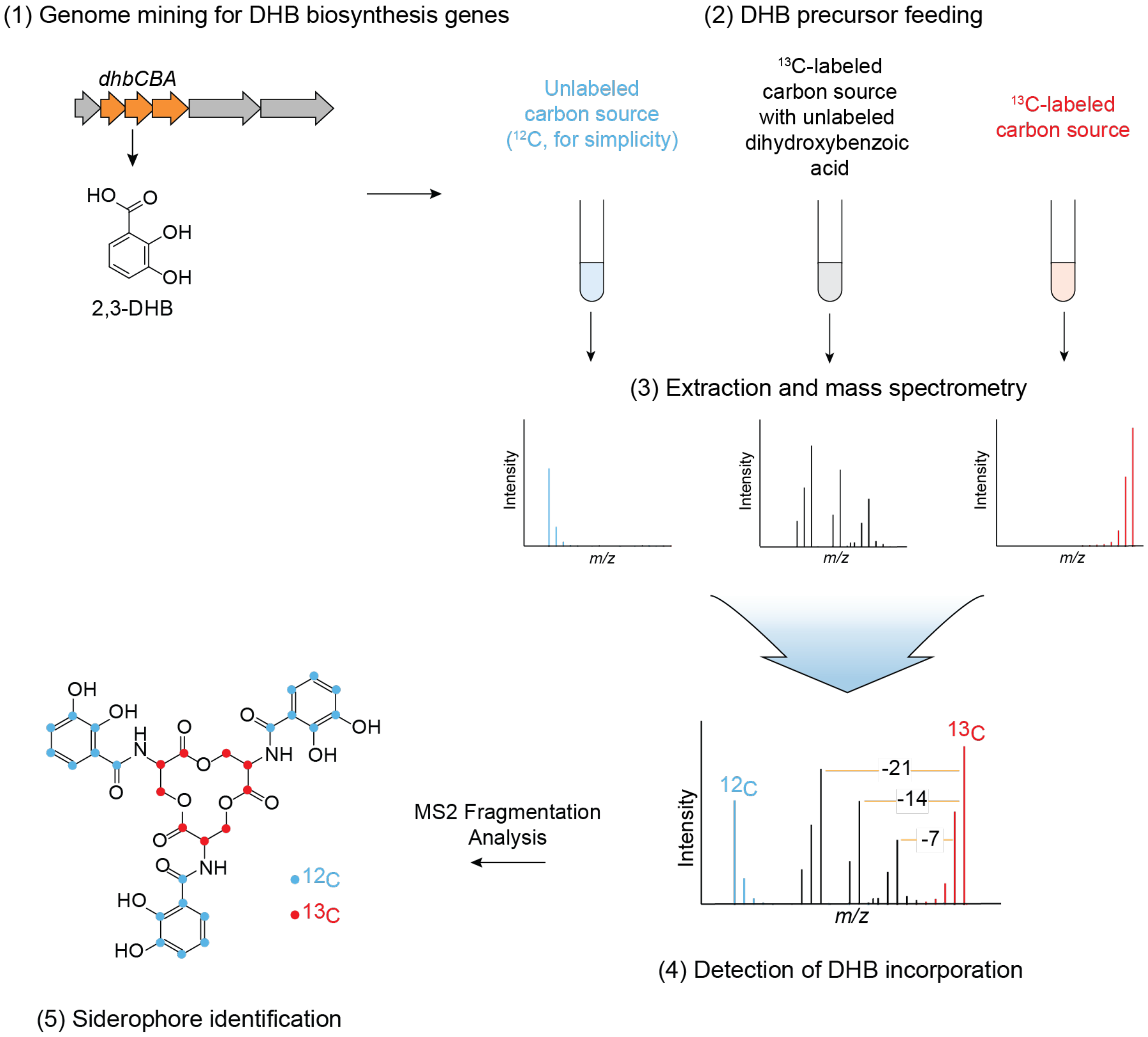

Figure S1. Scheme of the InverSIL experiment: (1) Genome mining for DHB biosynthesis genes. (2) The bacteria is grown in three different conditions: unlabeled carbon source [indicated as ^{12}C, for simplicity], ^{13}C-substituted carbon source [indicated as (^{13}C)] with addition of unlabeled 2,3-DHB, and ^{13}C-labeled carbon source. (3) Extraction and mass spectrometry. (4) High-resolution mass spectrometry to detect DHB incorporation. (5) Identification of the siderophore aided by MS2 fragmentation. The siderophore enterobactin is shown as an example.

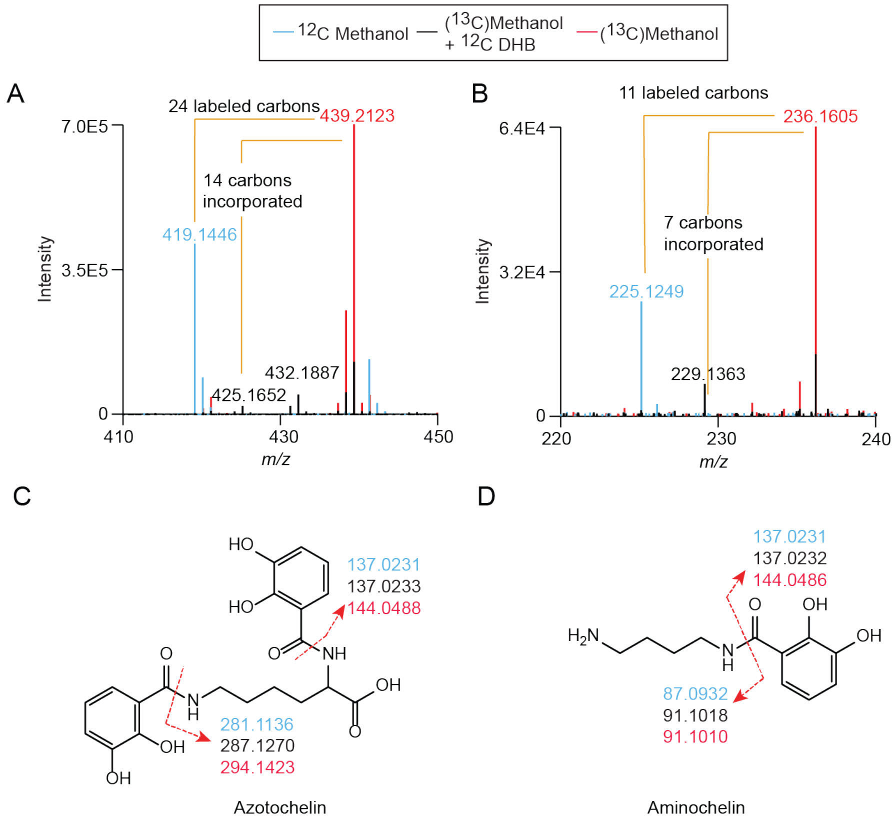

Figure S2. *Methylophilus* sp. strain 5 InverSIL showing incorporation of 2,3-DHB into (A) azotochelin and (B) aminochelin. (C) Structure of azotochelin with fragmentation data from different growth conditions. (D) Structure of aminochelin with fragmentation data from different growth conditions. The colors match the growth conditions indicated at the top of the figure.

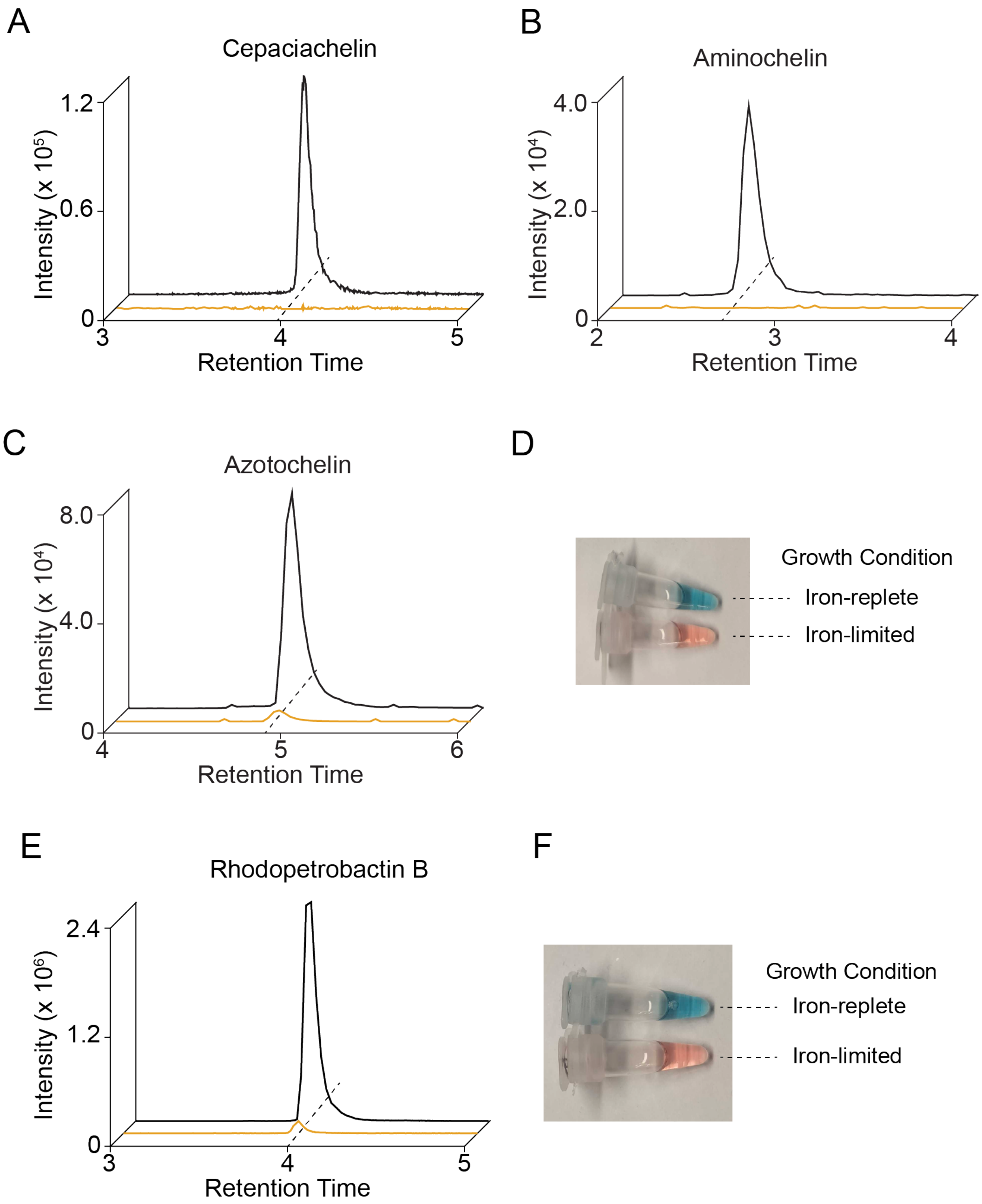

Figure S3. Siderophore production is induced by iron limitation in the methylotrophs *Methylophilus* sp. strain 5 and *Methylorubrum extorquens* PA1. (A) Extracted ion chromatograms for cepaciachelin (*m/z* 489.2340) of supernatant extracts of *Methylophilus* sp. strain 5 grown in iron-replete (yellow) and iron-limited (black) conditions. (B) Extracted ion chromatograms for aminochelin (*m/z* 225.1249) of supernatant extracts of *Methylophilus* sp. strain 5 grown in iron-replete (yellow) and iron-limited (black) conditions. (C) Extracted ion chromatograms for azotochelin (*m/z* 419.1446) of supernatant extracts of *Methylophilus* sp. strain 5 grown in iron-replete (yellow) and iron-limited (black) conditions. (D) Chrome azurol S (CAS) assay of supernatant extracts of *Methylophilus* sp. strain 5 grown in iron-replete and iron-limited conditions. (E) Extracted ion chromatograms for rhodopetrobactin B (*m/z* 831.4228) of supernatant extracts of *M. extorquens* PA1 grown in iron-replete (yellow) and iron-limited (black) conditions. (F) CAS

assay of supernatant extracts of *M. extorquens* PA1 grown in iron-replete and iron-limited conditions. For extracted ion chromatograms, mass tolerance < 5ppm. For CAS assays, a change of color from blue to brown indicates the presence of a siderophore in the supernatant extract.

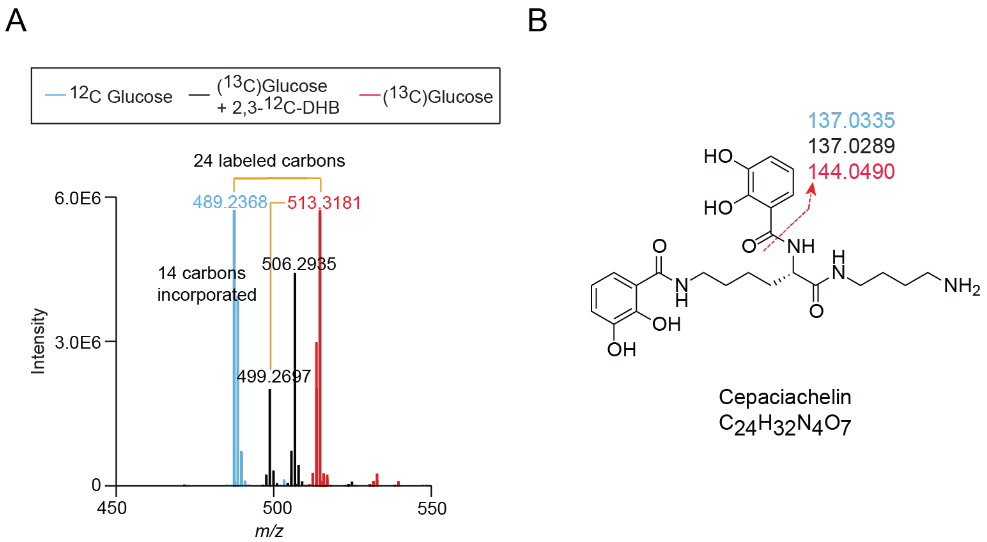

Figure S4. Using InverSIL to determine the structure of the chromobactin BGC product from *C. violaceum* CV017. (A) Overlayed mass spectra of *C. violaceum* CV017 supernatant extract showing incorporation of two 2,3-DHB units into a metabolite with the same high-resolution mass and carbon count as cepaciachelin. (B) Structure of chromobactin (cepaciachelin) showing MS2 fragments from different InverSIL conditions. The colors match the growth conditions indicated in panel A.

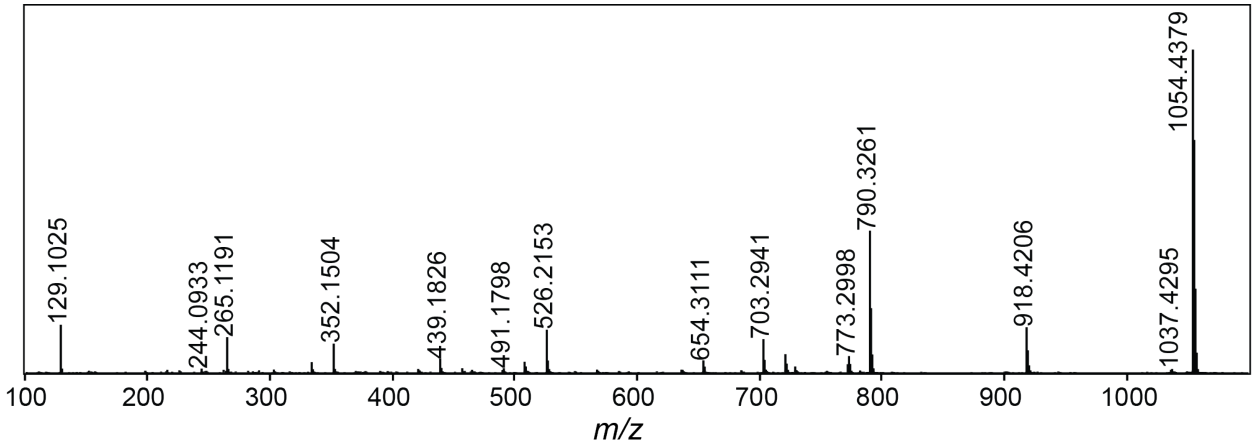

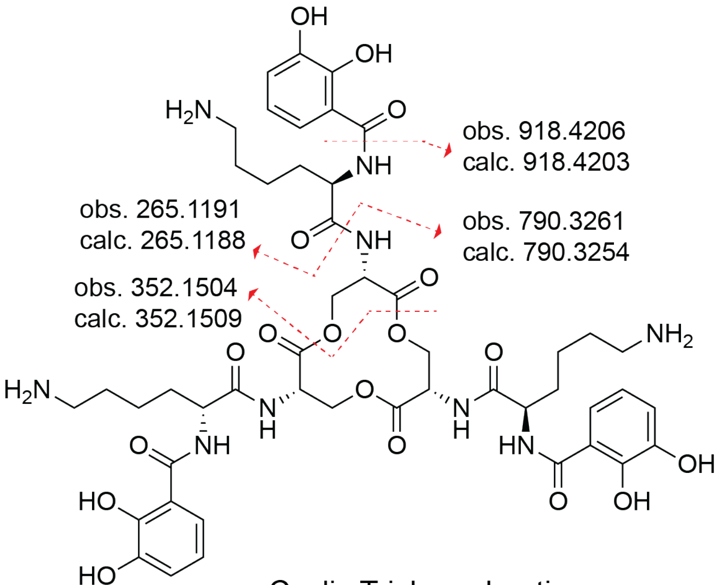

Figure S5. Fragmentation of cyclic trichrysobactin in *C. violaceum* CV017.

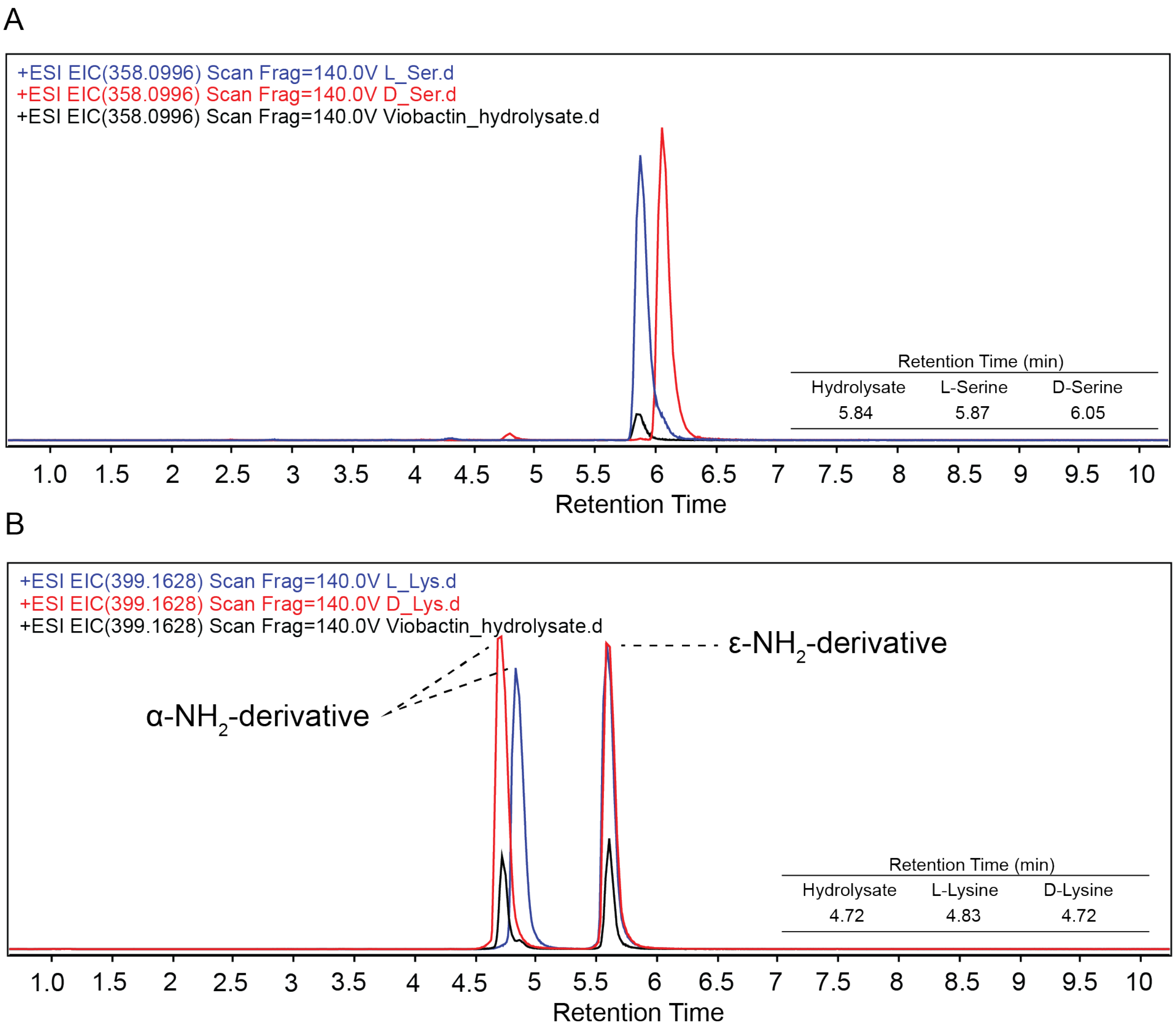

Figure S6. Amino acid stereochemistry determination of viobactin using Marfey's analysis. (A) Extracted ion chromatogram for *m/z* 358.0996 corresponding to $[M+H]^+$ of Marfey's derivatized serine. Blue (Marfey's derivatized L-serine), red (Marfey's derivatized D-serine), and black (Marfey's derivatized viobactin hydrolysate). Mass tolerance < 5ppm. (B) Extracted ion chromatogram for *m/z* 399.1628 corresponding to $[M+H]^+$ of Marfey's derivatized lysine. Blue (Marfey's derivatized L-lysine), red (Marfey's derivatized D-lysine), and black (Marfey's derivatized viobactin hydrolysate). Mass tolerance < 5ppm.

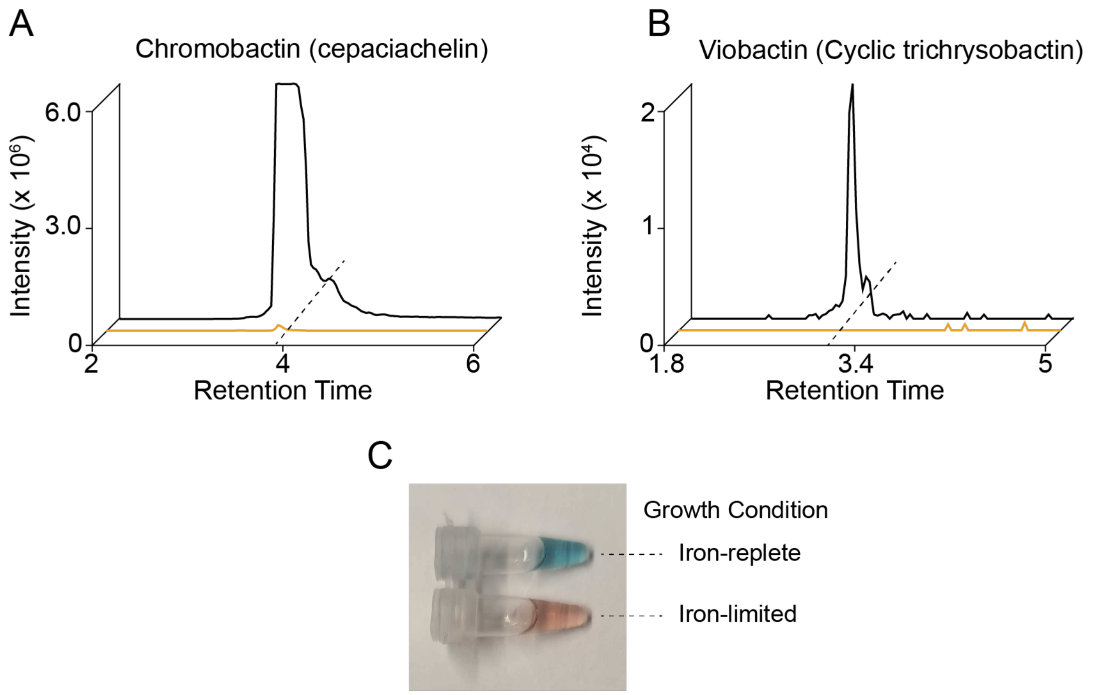

Figure S7. Chromobactin and viobactin production is induced by iron limitation. (A) Extracted ion chromatograms for chromobactin (*m/z* 489.2340) of supernatant extracts of *Chromobacter violaceum* CV017 grown in iron-replete (yellow) and iron-limited (black) conditions. (B) Extracted ion chromatograms for viobactin (*m/z* 1054.4379) of supernatant extracts of *Chromobacter violaceum* CV017 grown in iron-replete (yellow) and iron-limited (black) conditions. (C) CAS assay of supernatant extracts of *Chromobacter violaceum* CV017 grown in iron-replete and iron-limited conditions. For extracted ion chromatograms, mass tolerance < 5ppm. For the CAS assay, a change of color from blue to brown indicates the presence of a siderophore in the supernatant extract.

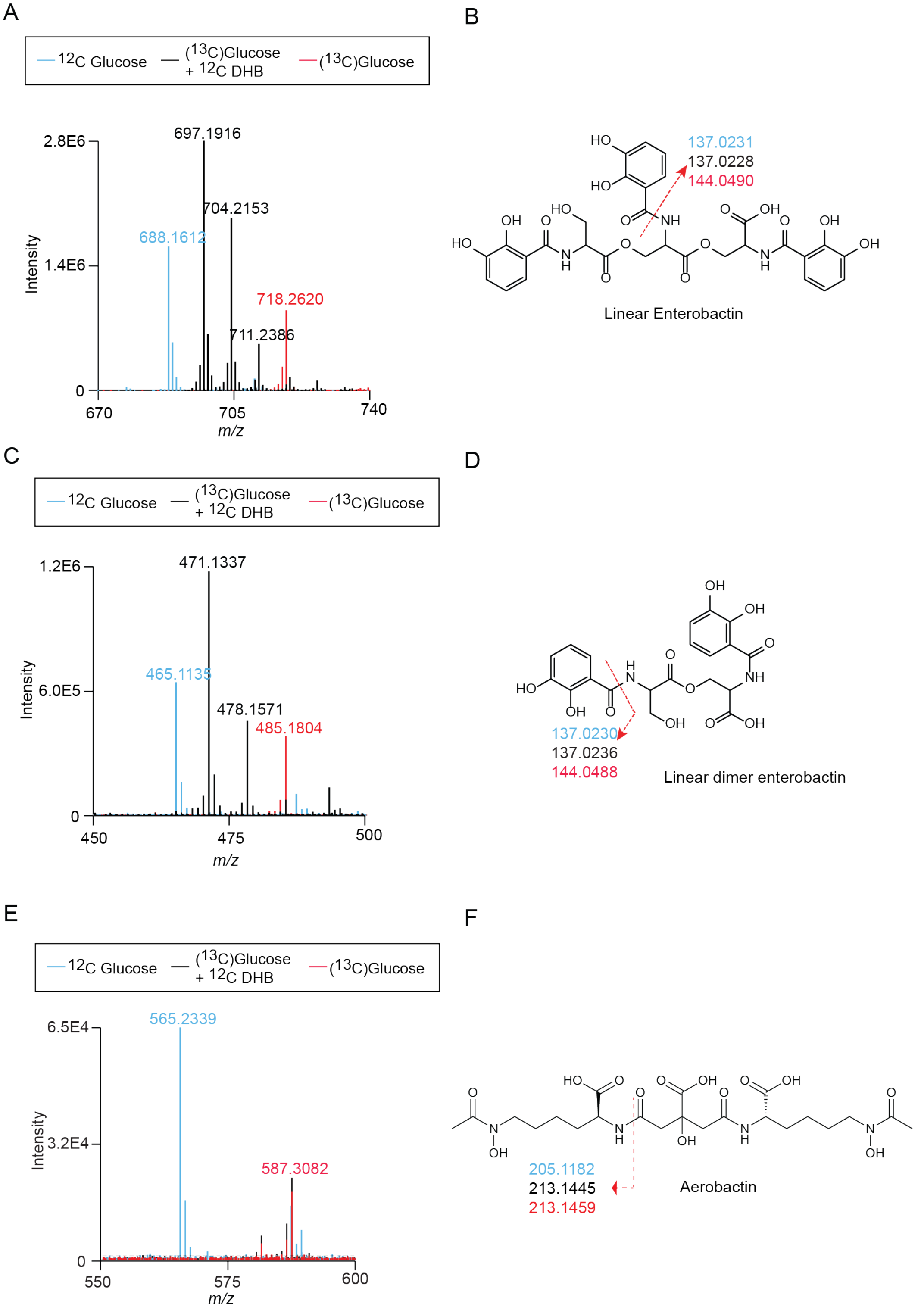

Figure S8. Other siderophores found in in *Kushneria konosiri* JCM16805. (A, C) Detection of other enterobactin forms in *Kushneria konosiri* JCM16805 using InverSIL. ^{12}C glucose (cyan), (^{13}C)glucose (red), (^{13}C)glucose + 2,3DHB (black) (B, D) Structure and MS2 fragmentation of the detected enterobactin forms. (E, F) Mass spectrum and structure of aerobactin in *Kushneria konosiri* JCM16805. ^{12}C glucose (cyan), (^{13}C)glucose (red), (^{13}C)glucose + 2,3DHB (black).

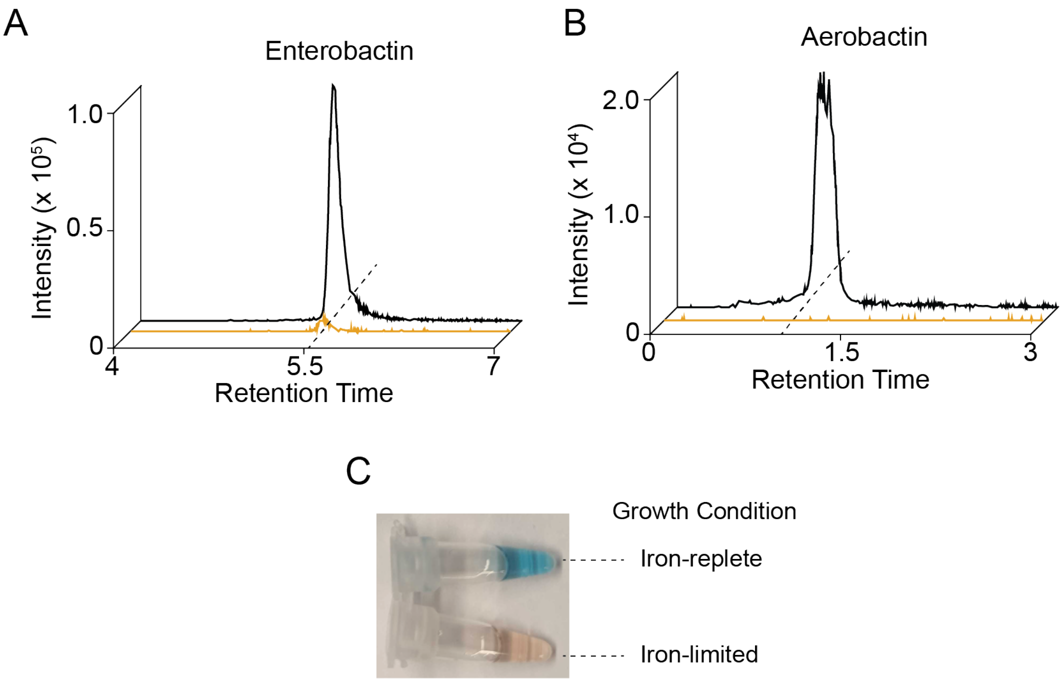

Figure S9. Catecholate siderophore production in *Kushneria konosiri* JCM16805 is induced by iron limitation. (A) Extracted ion chromatograms for enterobactin (*m/z* 670.1502) of supernatant extracts of *K. konosiri* JCM16805 grown in iron-replete (yellow) and iron-limited (black) conditions. (A) Extracted ion chromatograms for aerobactin (*m/z* 565.2339) of supernatant extracts of *K. konosiri* JCM16805 grown in iron-replete (yellow) and iron-limited (black) conditions. (C) CAS assay of supernatant extracts of *K. konosiri* JCM16805 grown in iron-replete and iron-limited conditions. For extracted ion chromatograms, mass tolerance < 5ppm. For the CAS assay, a change of color from blue to brown indicates the presence of a siderophore in the supernatant extract.

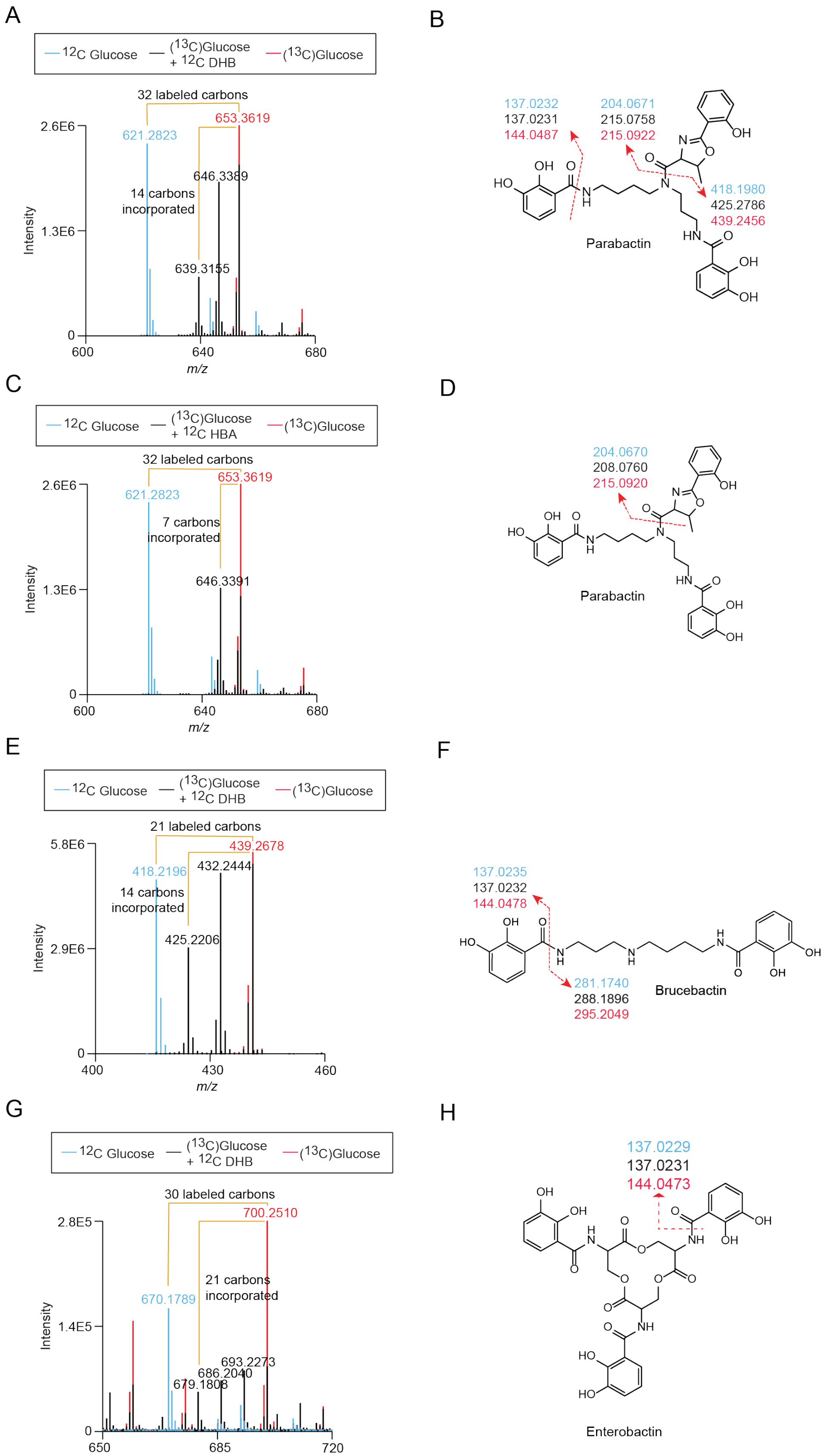
A
12C Glucose
(13C)Glucose + 12C DHB
(13C)Glucose
32 labeled carbons
621.2823
653.3619
646.3389
14 carbons incorporated
639.3155
Intensity
2.6E6
1.3E6
0
600
640
680
m/z
B
137.0232
137.0231
144.0487
204.0671
215.0758
215.0922
418.1980
425.2786
439.2456
Parabactin
C
12C Glucose
(13C)Glucose + 12C HBA
(13C)Glucose
32 labeled carbons
621.2823
653.3619
7 carbons incorporated
646.3391
Intensity
2.6E6
1.3E6
0
600
640
680
m/z
D
204.0670
208.0760
215.0920
Parabactin
E
12C Glucose
(13C)Glucose + 12C DHB
(13C)Glucose
21 labeled carbons
439.2678
418.2196
432.2444
14 carbons incorporated
425.2206
Intensity
5.8E6
2.9E6
0
400
430
460
m/z
F
137.0235
137.0232
144.0478
281.1740
288.1896
295.2049
Brucebactin
G
12C Glucose
(13C)Glucose + 12C DHB
(13C)Glucose
30 labeled carbons
700.2510
670.1789
21 carbons incorporated
693.2273
686.2040
679.1808
Intensity
2.8E5
1.4E5
0
650
685
720
m/z
H
137.0229
137.0231
144.0473
Enterobactin

Figure S10. Detection and inverse labeling of catecholate siderophores found in *Paracoccus denitrificans* PD1222. (A) Inverse labeling of parabactin using 2,3-DHB. (B) Structure of parabactin showing MS2 fragments from different InverSIL conditions. The colors match the growth conditions indicated in panel A. (C) Inverse labeling of parabactin using 2-hydroxybenzoic acid (HBA). (D) Structure of parabactin showing MS2 fragments from different InverSIL conditions. The colors match the growth conditions indicated in panel C. (E) Inverse labeling of brucebactin using 2,3-DHB. (F) Structure of brucebactin showing MS2 fragments of brucebactin from different InverSIL conditions. The colors match the growth conditions indicated in panel E. (G) Inverse labeling of enterobactin using 2,3-DHB. (H) Structure of enterobactin showing MS2 fragments of enterobactin from different InverSIL conditions. The colors match the growth conditions indicated in panel G.

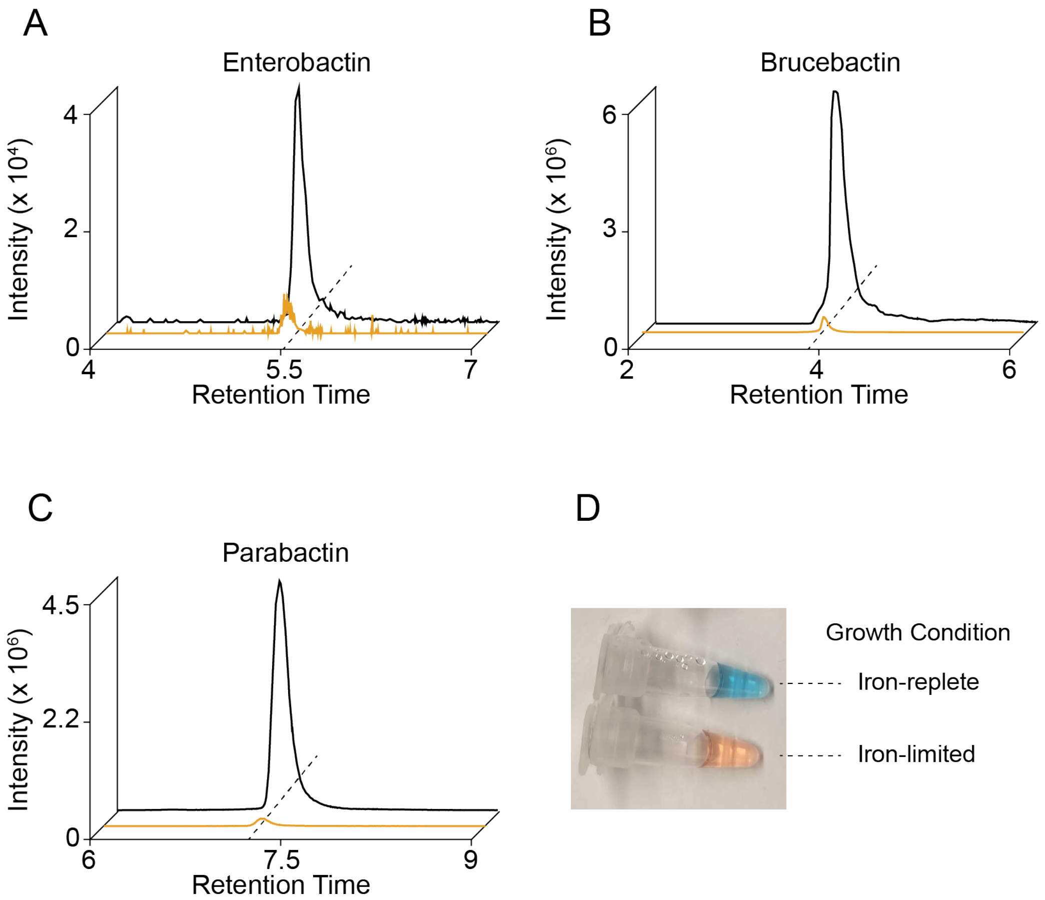

Figure S11. Catecholate siderophore production in *Paracoccus denitrificans* PD1222 is induced by iron limitation. (A) Extracted ion chromatograms for enterobactin (*m/z* 670.1502) of supernatant extracts of *P. denitrificans* PD1222 grown in iron-replete (yellow) and iron-limited conditions (black). (B) Extracted ion chromatograms for brucebactin (*m/z* 418.2196) of supernatant extracts of *P. denitrificans* PD1222 grown in iron-replete (yellow) and iron-limited (black) conditions. (C) Extracted ion chromatograms for parabactin (*m/z* 621.2823) of supernatant extracts of *P. denitrificans* PD1222 grown in iron-replete (yellow) and iron-limited (black) conditions. (D) CAS assay of supernatant extracts of *P. denitrificans* PD1222 grown in iron-replete and iron-limited conditions. For extracted ion chromatograms, mass tolerance < 5ppm. For the CAS assay, a change of color from blue to brown indicates the presence of a siderophore in the supernatant extract.

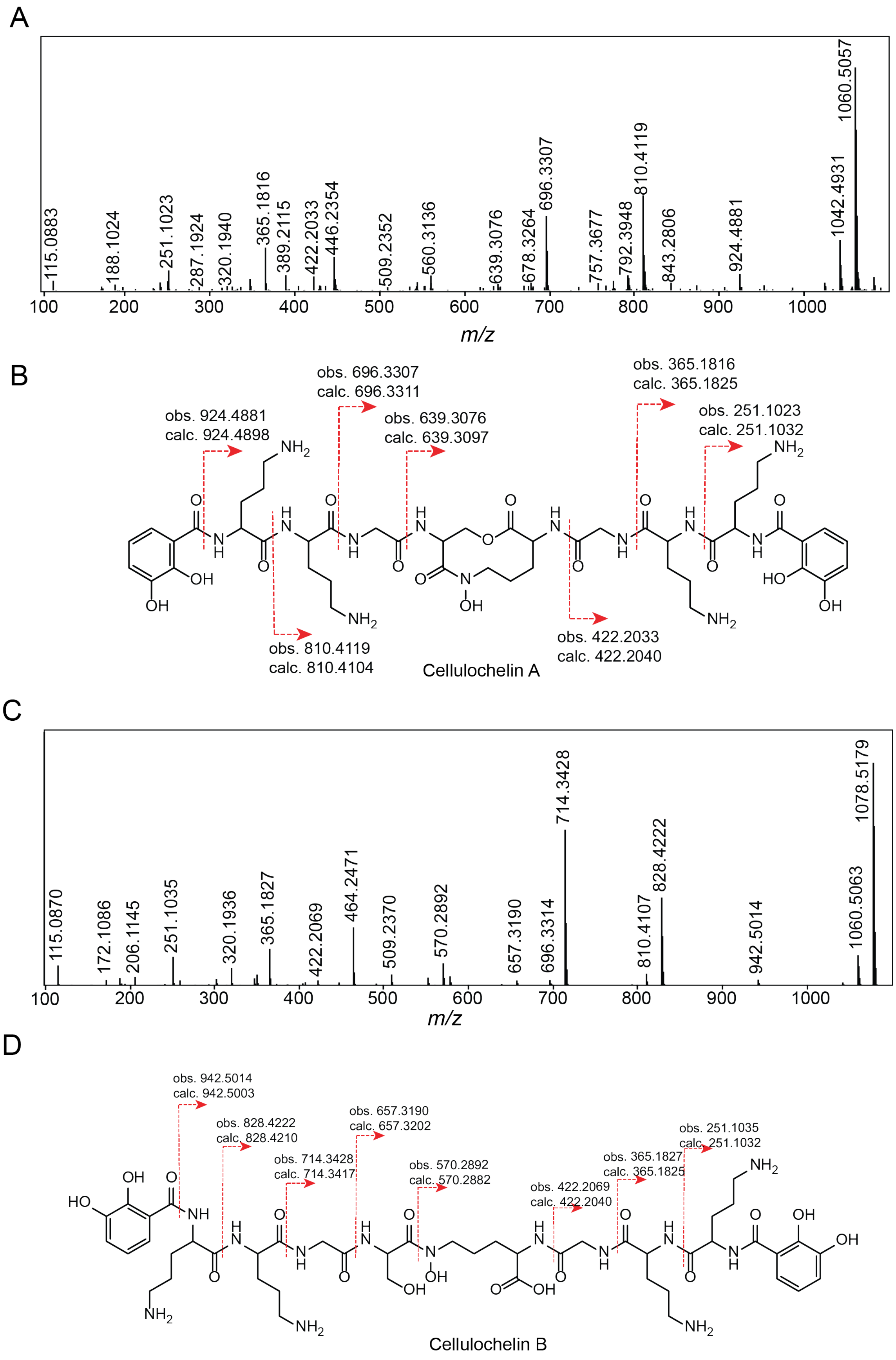
A
115.0883
188.1024
251.1023
287.1924
320.1940
365.1816
389.2115
422.2033
446.2354
509.2352
560.3136
639.3076
678.3264
696.3307
757.3677
792.3948
810.4119
843.2806
924.4881
1042.4931
1060.5057
m/z
B
obs. 924.4881
calc. 924.4898
obs. 696.3307
calc. 696.3311
obs. 639.3076
calc. 639.3097
obs. 365.1816
calc. 365.1825
obs. 251.1023
calc. 251.1032
obs. 810.4119
calc. 810.4104
obs. 422.2033
calc. 422.2040
Cellulochelin A
C
115.0870
172.1086
206.1145
251.1035
320.1936
365.1827
422.2069
464.2471
509.2370
570.2892
657.3190
696.3314
714.3428
810.4107
828.4222
942.5014
1060.5063
1078.5179
m/z
D
obs. 942.5014
calc. 942.5003
obs. 828.4222
calc. 828.4210
obs. 714.3428
calc. 714.3417
obs. 657.3190
calc. 657.3202
obs. 570.2892
calc. 570.2882
obs. 422.2069
calc. 422.2040
obs. 365.1827
calc. 365.1825
obs. 251.1035
calc. 251.1032
Cellulochelin B

Figure S12. MS2 fragmentation of cellulochelin A and B. (A, B) Cellulochelin A MS2 fragmentation and structure of the parent ion 1060.5057 *m/z*. (C, D) Cellulochelin B MS2 fragmentation and structure of the parent ion 1078.5179 *m/z*.

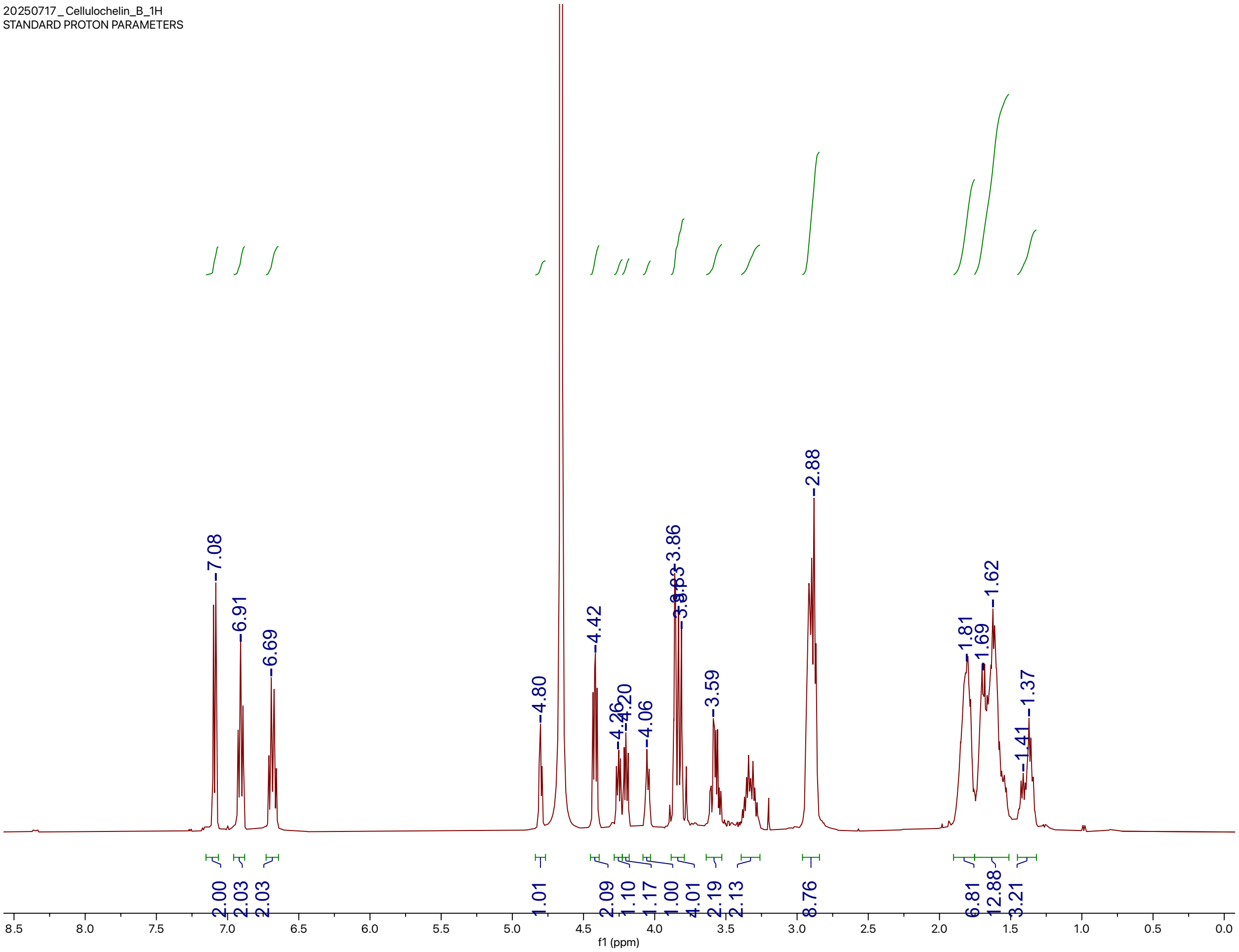

Figure S13. ^{1}H NMR spectrum of cellulochelin B in D_2O (500 MHz)**.** Summarized in Table S4.

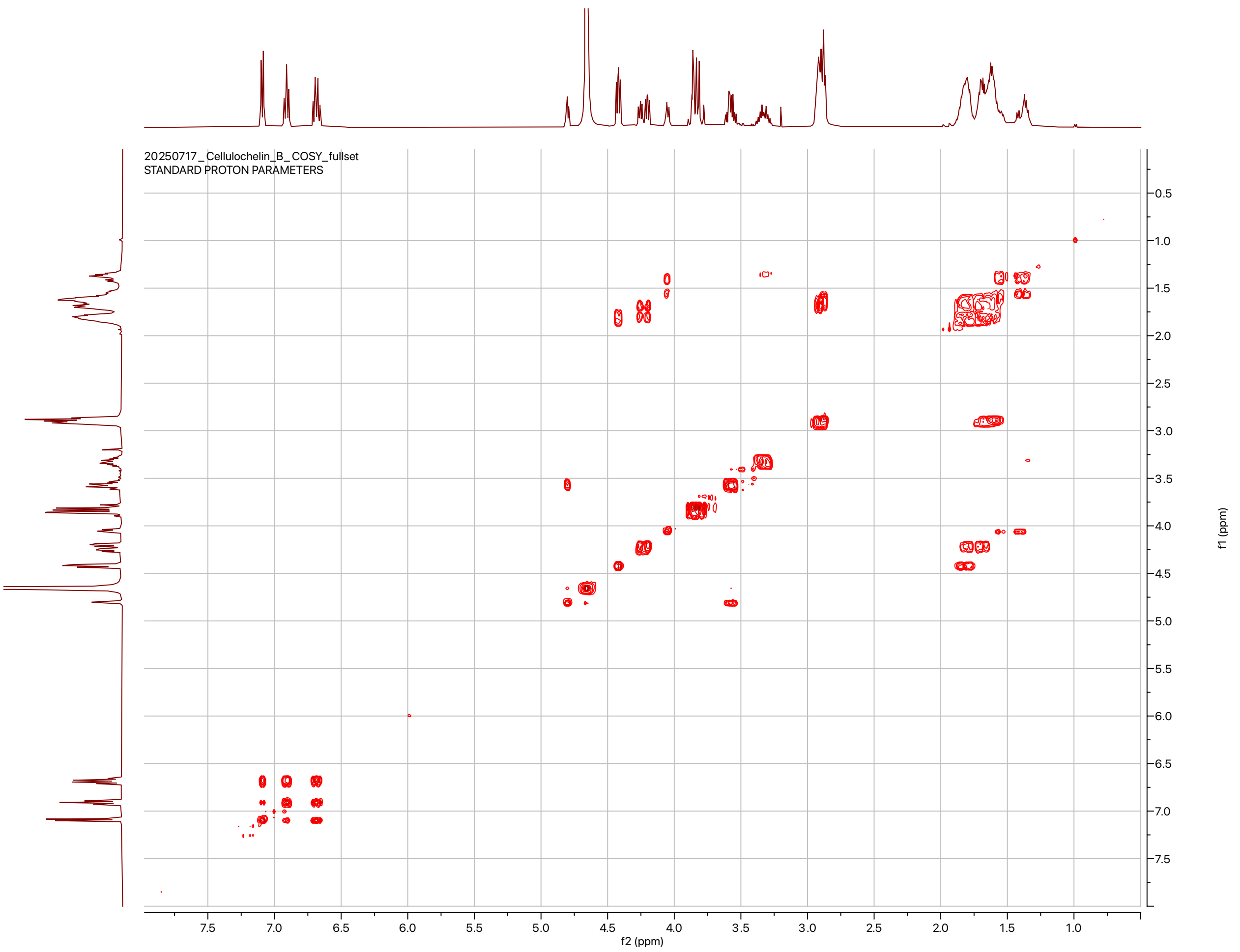

Figure S14. COSY NMR spectrum of cellulochelin B in D_2O (500 MHz)**.** Summarized in Table S4.

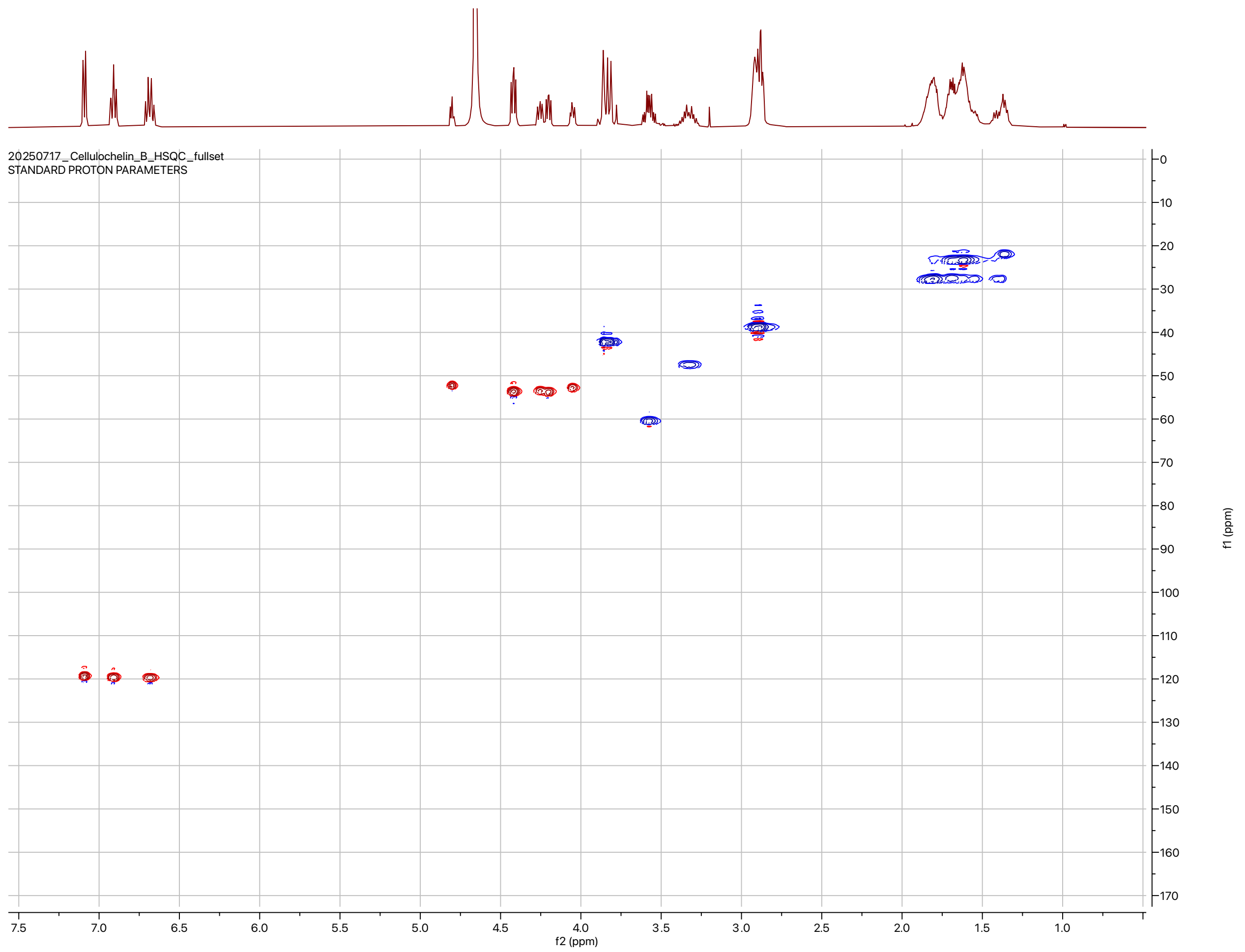

Figure S15. gHSQC spectrum of cellulochelin B in D_2O (500 MHz)**.** Summarized in Table S4.

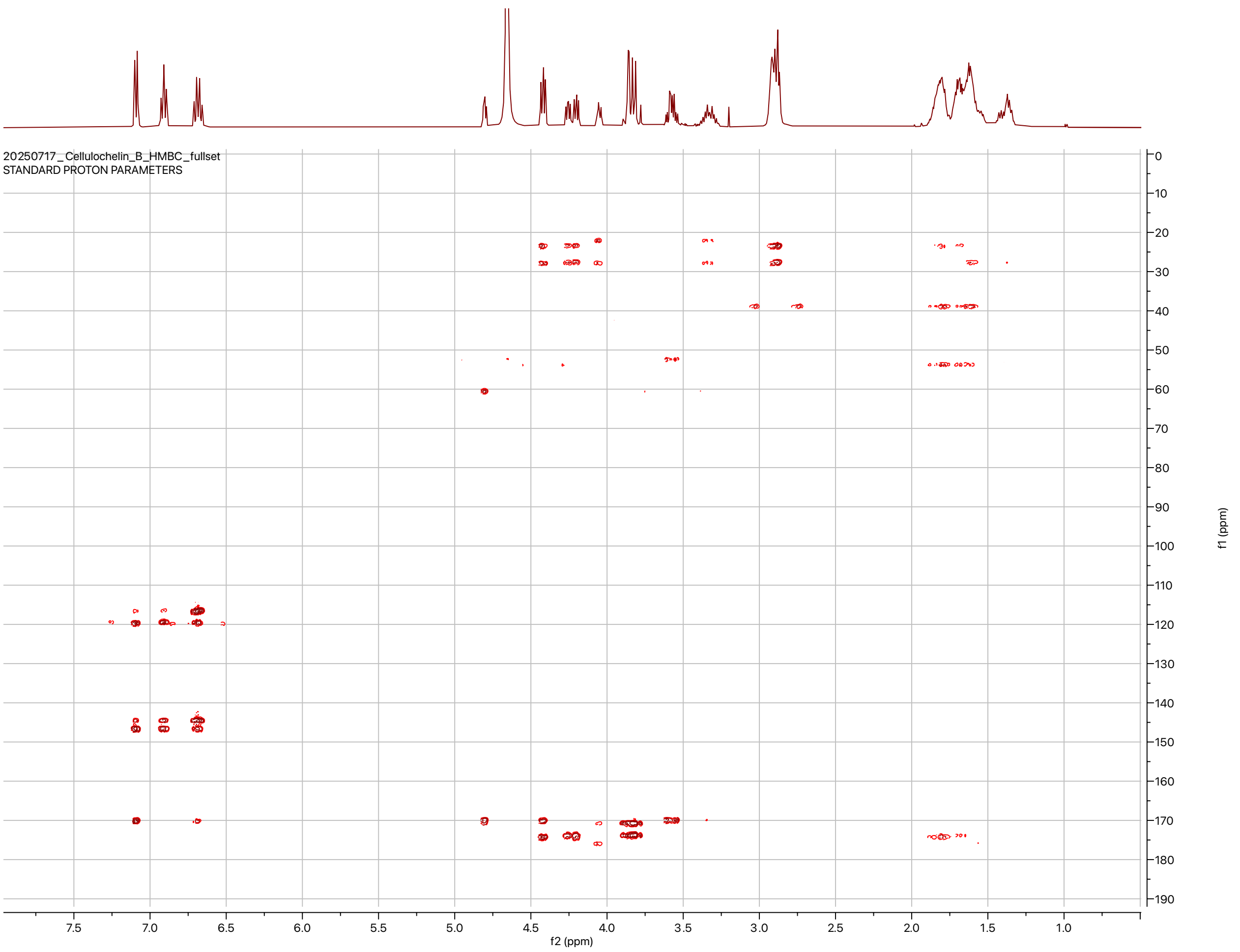

Figure S16. HMBC NMR spectrum of cellulochelin B in D_2O (500 MHz)**.** Summarized in Table S4.

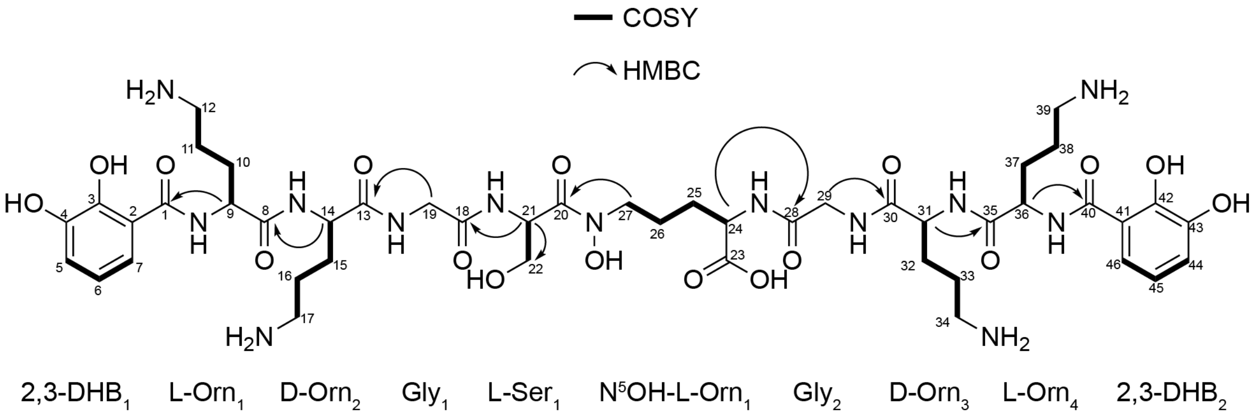

Figure S17. COSY and HMBC NMR correlations in cellulochelin B in D_2O (500 MHz)**.**

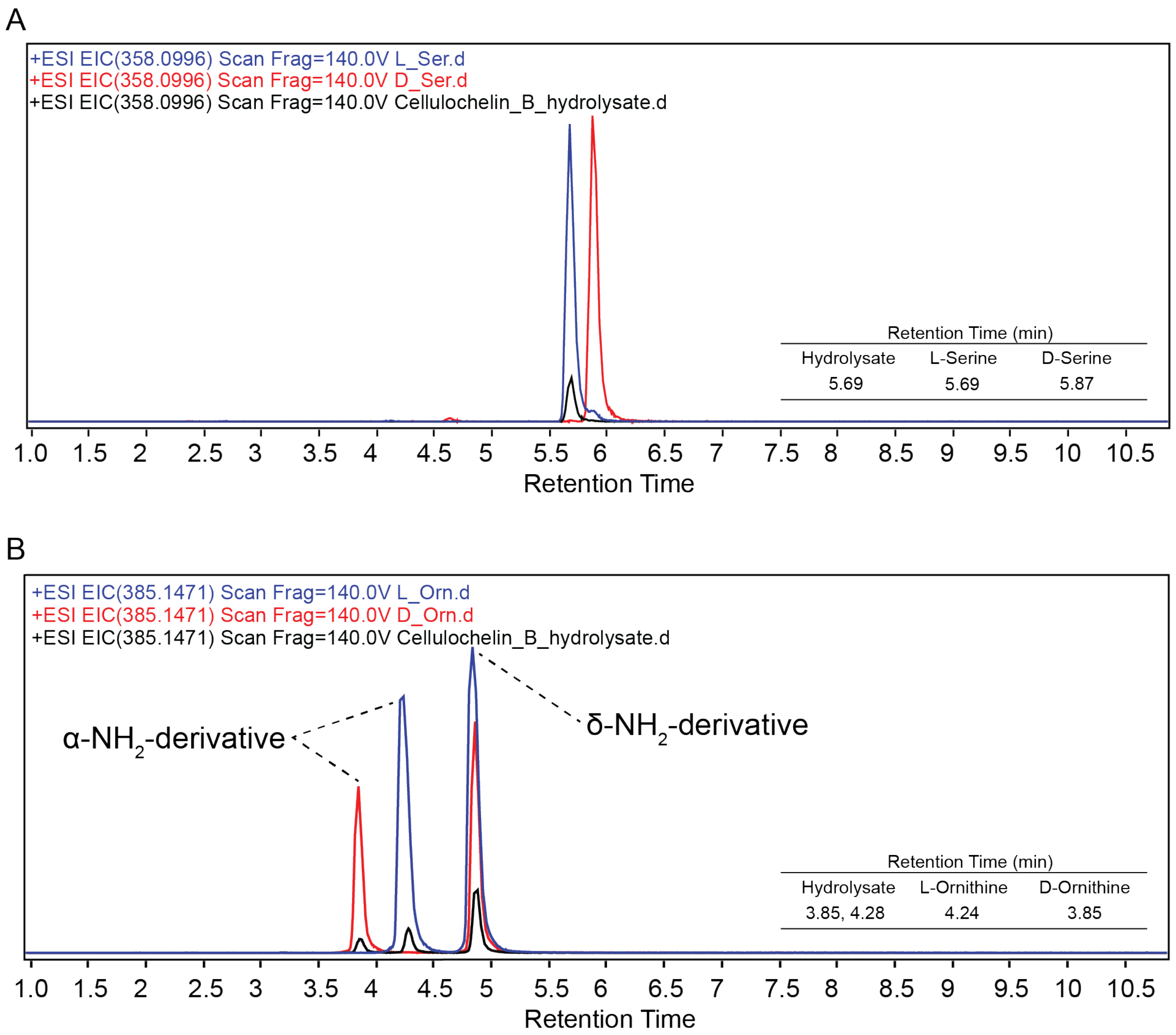

Figure S18. Amino acid stereochemistry determination of cellulochelin B using Marfey's analysis. (A) Extracted ion chromatogram for *m/z* 358.0996 corresponding to $[M+H]^+$ of Marfey's derivatized serine. Blue (Marfey's derivatized L-serine), red (Marfey's derivatized D-serine), and black (Marfey's derivatized cellulochelin B hydrolysate). Mass tolerance < 5ppm. (B) Extracted ion chromatogram for *m/z* 385.1471 corresponding to $[M+H]^+$ of Marfey's derivatized ornithine. Blue (Marfey's derivatized L-ornithine), red (Marfey's derivatized D-ornithine), and black (Marfey's derivatized cellulochelin B hydrolysate) Mass tolerance < 5ppm.

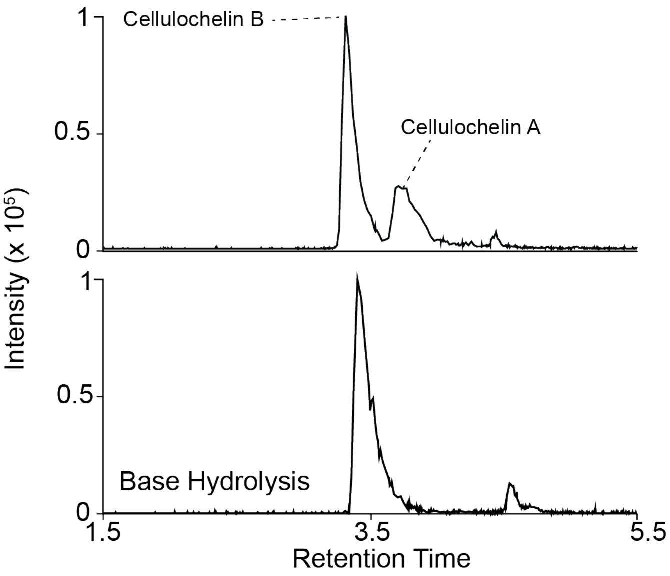

Figure S19. Cellulochelin A is converted into cellulochelin B via degradation of the cellulochelin A macrolactone by base hydrolysis. Combined extracted base peak chromatograms of supernatant extracts from *Cellulomonas* sp. strain Leaf 334 for cellulochelin A (*m/z* 1060.5057) and cellulochelin B (*m/z* 1078.5179) before (top) and after (bottom) base hydrolysis. Mass tolerance < 5ppm.

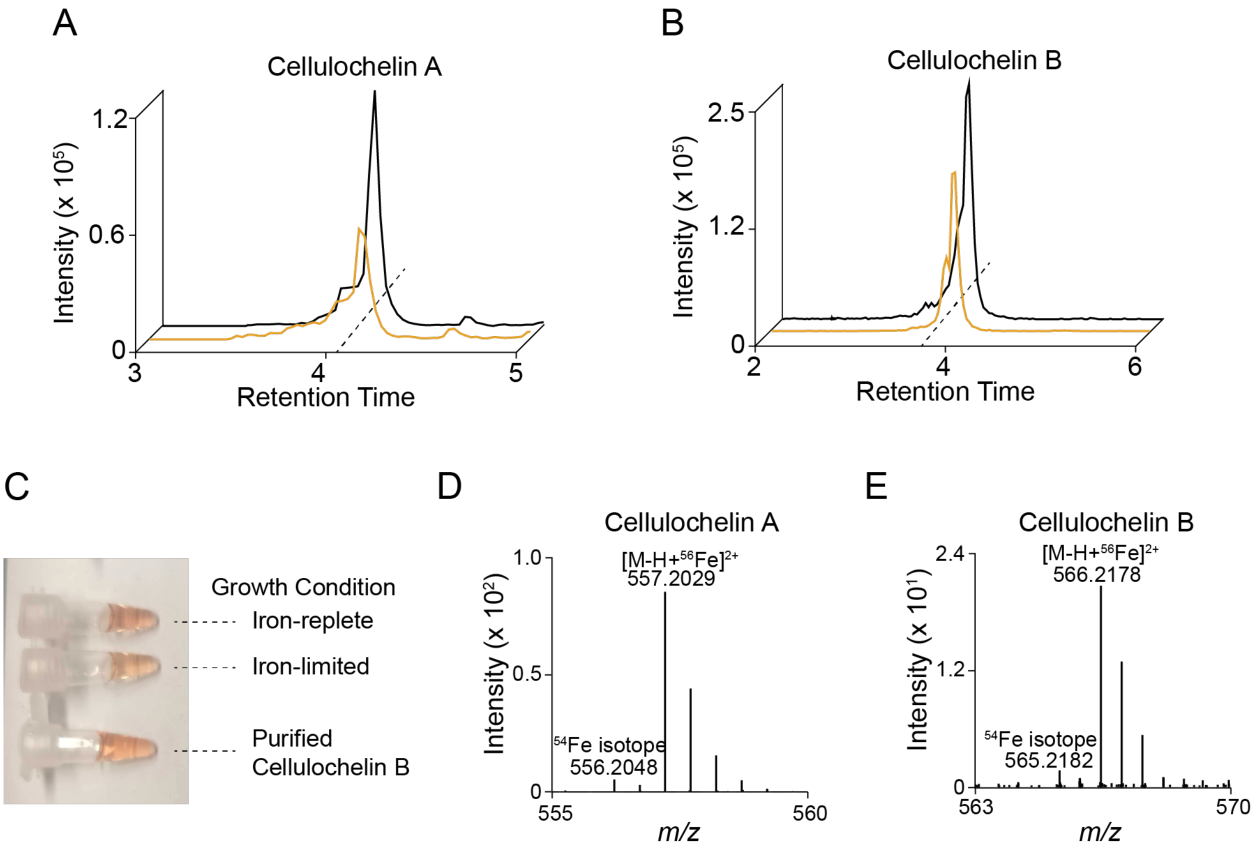

Figure S20. Cellulochelin A and B are produced constitutively by *Cellulomonas* sp. strain Leaf 334. (A) Extracted ion chromatograms for cellulochelin A (*m/z* 1060.5057) of supernatant extracts of *Cellulomonas* sp. strain Leaf334 grown in iron-replete (yellow) and iron-limited (black) conditions. (B) Extracted ion chromatograms for cellulochelin B (*m/z* 1078.5179) of supernatant extracts of *Cellulomonas* sp. strain Leaf334 grown in iron-replete (yellow) and iron-limited (black) conditions. (C) CAS assay of supernatant extracts of *Cellulomonas* sp. strain Leaf334 grown in iron-replete and iron-limited conditions and of purified cellulochelin B. A change of color from blue to brown indicates the presence of a siderophore in the supernatant extract. (D) ^{54}Fe-^{56}Fe isotope pattern of the Fe-cellulochelin A complex. (E) ^{54}Fe-^{56}Fe isotope pattern of the Fe-cellulochelin B complex. For extracted ion chromatograms, mass tolerance < 5ppm.

SUPPLEMENTARY TABLES

Table S1. Cepaciachelin MS/MS fragmentation comparison between crude extracts from *B. ambifaria* BAA244, *Methylophilus* sp. strain 5, and *C. violaceum* CV017.

Burkholderia ambifaria BAA244		*Methylophilus* sp. strain 5		*Chromobacterium violaceum* CV017	
m/z	Intensity	*m/z*	Intensity	*m/z*	Intensity
84.0903	3.5E+06	84.0805	2.5E+06	84.0809	3.5E+05
72.0896	2.6E+06	72.0804	1.8E+06	72.0809	2.4E+05
89.1171	1.7E+06	89.1069	1.2E+06	89.1074	1.4E+05
220.1124	1.5E+06	220.0967	1.1E+06	220.097	1.3E+05
353.2379	7.3E+05	353.2185	5.7E+05	353.2186	6.1E+04
217.2178	2.8E+05	217.2023	1.7E+05	217.2025	2.4E+04
85.0937	2.4E+05	85.0837	1.5E+05	221.1004	2.2E+04
221.1158	2.4E+05	129.1021	1.1E+05	85.0842	2.1E+04
129.1141	1.7E+05	354.2218	1.1E+05	354.2218	2.0E+04
137.0355	1.7E+05	137.0231	9.9E+04	137.0234	1.6E+04

Table S2. Rhodopetrobactin B MS/MS fragmentation comparison between crude extracts from *R. palustris* CGA009 and *M. extorquens* PA1.

Rhodopseudomonas palustris CGA009		*Methylorubrum extorquens* PA1	
m/z	Intensity	*m/z*	Intensity
338.2078	1.0E+05	338.2084	1.2E+04
202.1913	1.0E+05	202.192	1.2E+04
677.3871	6.4E+04	677.3878	8.1E+03
541.3709	5.7E+04	541.3713	7.5E+03
312.1919	4.0E+04	312.1924	5.0E+03
185.1649	3.7E+04	185.1651	4.2E+03
321.1813	3.4E+04	321.182	3.8E+03
678.3909	2.7E+04	678.3914	2.7E+03
208.0971	2.2E+04	358.1978	2.6E+03
482.3345	2.1E+04	208.0979	2.4E+03

Table S3. Enterobactin MS/MS fragmentation comparison between crude extracts from *E. coli* MG1655, *K. konosiri* JCM16805, and *P. denitrificans* PD1222.

Escherichia coli MG1655		*Kushneria konosiri* JCM16805		*Paracoccus denitrificans* PD1222	
m/z	Intensity	*m/z*	Intensity	*m/z*	Intensity
224.0552	2.3E+05	224.0552	2.0E+05	224.0555	3.8E+05
225.0584	2.9E+04	178.0493	2.9E+04	225.0585	4.9E+04
206.0446	2.7E+04	137.0228	2.8E+04	447.103	1.1E+04
137.0233	1.7E+04	225.0581	2.5E+04	206.0444	9.9E+03
447.1027	1.0E+04	150.0546	8.9E+03	311.0869	9.6E+03
196.06	7.9E+03	206.0445	7.3E+03	178.0498	8.7E+03
178.0495	6.5E+03	157.0608	4.2E+03	196.0606	7.6E+03
157.0606	5.4E+03	179.0528	4.1E+03	137.0234	7.3E+03
226.0591	4.4E+03	162.0553	3.7E+03	226.0596	6.8E+03
293.0765	4.3E+03	60.0439	3.1E+03	448.1067	4.1E+03

Table S4. Summary of ^{1}H NMR data (δ in ppm) and ^{13}C NMR data (δ in ppm) for cellulochelin B in D_2O.

Residue	Position	Type	δC	δH, multiplicity (J in Hz)	HMBC	COSY
2,3-Dihydroxy benzoic acid (1)	1	C=O	170	-	-	-
	2	qC	116.5	-	-	-
	3	C	146.4	-	-	-
	4	C	144.3	-	-	-
	5	CH	119.6	6.91, t (8.65)	3, 4, 6, 7	6, 7
	6	CH	119.6	6.69, d (8.07)	1, 2, 4, 5, 6	5, 7
	7	CH	119.3	7.08, m	1, 2, 3, 5, 6	5, 6
D-Ornithine (1)	8	C=O	174.1	-	-	-
	9	α-CH	53.6	4.42, dd (5.80, 8.74)	1, 10, 11	10
	10	β-CH2	27.7	1.81, 1.84, m	9, 11, 12	9, 11
	11	γ-CH2	24.03	1.65, 1.67, m	9, 10, 12	10, 12
	12	δ-CH2	38.6	2.88, 2.90, m	10, 11	11
L-Ornithine (2)	13	C=O	173.7	-	-	-
	14	α-CH	53.4	4.26, dd (5.63, 8.65)	8, 13, 15, 16	15
	15	β-CH2	27.5	1.79, 1.81, m	14, 16, 17	14, 16
	16	γ-CH2	23.1	1.70, 1.69, m	14, 15, 17	15, 17
	17	δ-CH2	38.6	2.88, 2.90, m	15, 16	16
Glycine (1)	18	C=O	170.7	-	-	-
	19	α-CH2	42.1	3.86, 3.85	13, 18	
L-Serine	20	C=O	169.8	-	-	-
	21	α-CH	52.3	4.8, t (5.21)	18, 19, 20, 22	22
	22	β-CH2	60.3	3.59, m	20, 21	21
L-N-OH-Orn	23	COOH	175.7	-	-	-
	24	α-CH	52.7	4.06, m	23, 25, 26, 28	25
	25	β-CH2	21.9	1.38, 1.41, m	24, 26, 27	24, 26
	26	γ-CH2	27.6	1.57, m	24, 25, 27	25, 27

	27	δ-CH2	47.4	3.34, 3.31, m	20, 25, 26	26
Glycine (2)	28	C=O	170.7	-	-	-
	29	CH2	42.1	3.78, 3.84	28, 30	
L-Ornithine (3)	30	C=O	173.7	-	-	-
	31	α-CH	53.6	4.20, dd (5.80, 8.40)	30, 32, 33, 35	32
	32	β-CH2	27.5	1.79, 1.81, m	31, 33, 34	31, 33
	33	γ-CH2	23.1	1.70, 1.69, m	31, 32, 34	32, 34
	34	δ-CH2	38.6	2.88, 2.90, m	32, 33	33
D-Ornithine (4)	35	C=O	174.1	-	-	-
	36	α-CH	53.6	4.42, dd (5.80, 8.74)	37, 38, 40	37
	37	β-CH2	27.7	1.81, 1.84, m	36, 38, 39	36, 38
	38	γ-CH2	24.03	1.65, 1.67, m	36, 37, 39	37, 39
	39	δ-CH2	38.6	2.88, 2.90, m	37, 38	38
2,3-Dihydroxy benzoic acid (2)	40	C=O	170	-	-	-
	41	qC	116.5	-	-	-
	42	C	146.4	-	-	-
	43	C	144.3	-	-	-
	44	CH	119.6	6.91, t (8.65)	42, 43, 45, 46	45, 46
	45	CH	119.6	6.69, d (8.07)	40, 41, 43, 44, 46	44, 46
	46	CH	119.3	7.08, m	40, 41, 42, 44, 45	44, 45

Table S5. List of strains used in the study.

Strain	Reference
Methylophilus sp. strain 5	(1)
Burkholderia ambifaria BAA-244	(2)
Chromobacterium violaceum CV017	(3, 4)
Methylorubrum extorquens PA1	(5, 6)
Rhodopseudomonas palustris CGA009	(7)
Escherichia coli MG1655	(8)
Kushneria konosiri JCM16805	(9, 10)
Paracoccus denitrificans PD1222	(11)
Cellulomonas sp. strain Leaf334	(12)

REFERENCES

1. Chistoserdova L. 2010. Genomes of fifty methylotrophs isolated from Lake Washington. DOE Joint Genome Institute.
2. Barelmann I, Meyer J-M, Taraz K, Budzikiewicz H. 1996. Cepaciachelin, A New Catecholate Siderophore From *Burkholderia* (*Pseudomonas*) *cepacia*. *Z Naturforsch C* 51:627–630. https://doi.org/10.1515/znc-1996-9-1004
3. Chernin LS, Winson MK, Thompson JM, Haran S, Bycroft BW, Chet I, Williams P, Stewart GSAB. 1998. Chitinolytic Activity in Chromobacterium violaceum: Substrate Analysis and Regulation by Quorum Sensing. *J Bacteriol* 180:4435–4441. https://doi.org/10.1128/jb.180.17.4435-4441.1998
4. Chandler JR, Heilmann S, Mittler JE, Greenberg EP. 2012. Acyl-homoserine lactone-dependent eavesdropping promotes competition in a laboratory co-culture model. ISME J 6:2219–2228. https://doi.org/10.1038/ismej.2012.69
5. Knief C, Dengler V, Bodelier PLE, Vorholt JA. 2012. Characterization of Methylobacterium strains isolated from the phyllosphere and description of Methylobacterium longum sp. nov. Antonie Van Leeuwenhoek 101:169–183. https://doi.org/10.1007/s10482-011-9650-6
6. Marx CJ, Bringel F, Chistoserdova L, Moulin L, Farhan Ul Haque M, Fleischman DE, Gruffaz C, Jourand P, Knief C, Lee M-C, Muller EEL, Nadalig T, Peyraud R, Roselli S, Russ L, Goodwin LA, Ivanova N, Kyrpides N, Lajus A, Land ML, Médigue C, Mikhailova N, Nolan M, Woyke T, Stolyar S, Vorholt JA, Vuilleumier S. 2012. Complete Genome Sequences of Six Strains of the Genus Methylobacterium. J Bacteriol 194:4746–4748. https://doi.org/10.1128/JB.01009-12
7. Baars O, Morel FMM, Zhang X. 2018. The purple non-sulfur bacterium *Rhodopseudomonas palustris* produces novel petrobactin-related siderophores under aerobic and anaerobic conditions. *Environ Microbiol* 20:1667–1676. https://doi.org/10.1111/1462-2920.14078
8. Edwards JS, Palsson BO. 2000. The *Escherichia coli* MG1655 *in silico* metabolic genotype: Its definition, characteristics, and capabilities. *Proc Natl Acad Sci USA* 97:5528–5533. https://doi.org/10.1073/pnas.97.10.5528
9. Navarro-Torre S, Carro L, Rodríguez-Llorente ID, Pajuelo E, Caviedes MÁ, Igual JM, Redondo-Gómez S, Camacho M, Klenk H-P, Montero-Calasanz MDC. 2018. *Kushneria phyllosphaerae* sp. nov. and *Kushneria endophytica* sp. nov., plant growth promoting endophytes isolated from the halophyte plant *Arthrocnemum macrostachyum*. *Int J Syst Evol Microbiol* 68:2800–2806. https://doi.org/10.1099/ijsem.0.002897
10. Meinzer M, Ahmad N, Nielsen BL. 2023. Halophilic Plant-Associated Bacteria with Plant-Growth-Promoting Potential. *Microorganisms* 11:2910. https://doi.org/10.3390/microorganisms11122910
11. De Vries GE, Harms N, Hoogendijk J, Stouthamer AH. 1989. Isolation and characterization of *Paracoccus denitrificans* mutants with increased conjugation frequencies and pleiotropic loss of a (nGATCn) DNA-modifying property. *Arch Microbiol* 152:52–57. https://doi.org/10.1007/BF00447011
12. Bai Y, Müller DB, Srinivas G, Garrido-Oter R, Potthoff E, Rott M, Dombrowski N, Münch PC, Spaepen S, Remus-Emsermann M, Hüttel B, McHardy AC, Vorholt JA, Schulze-Lefert P.

2015. Functional overlap of the *Arabidopsis* leaf and root microbiota. *Nature* 528:364–369. https://doi.org/10.1038/nature16192
